# Fabrication of Perovskite Film-Coated Hollow Capillary Fibers Using a Fast Solvent Exchange Method

**DOI:** 10.3390/nano11061483

**Published:** 2021-06-03

**Authors:** Xuesong Li, Pan Zeng, Qiongrong Ou, Shuyu Zhang

**Affiliations:** 1 Academy for Engineering and Technology, Fudan University, Shanghai 200433, China; 18210860009@fudan.edu.cn (X.L.); qrou@fudan.edu.cn (Q.O.); 2 Institute for Electric Light Sources, School of Information Science and Technology, Fudan University, Shanghai 200433, China; 16110720005@fudan.edu.cn

**Keywords:** perovskite film, capillary fiber, solvent exchange, amplified spontaneous emission

## Abstract

Metal halide perovskites have been successfully applied in a variety of fields such as LEDs, lasers and solar cells, thanks to their excellent optoelectronic properties. Capillary fibers can further expand the range of perovskite applications and at the same time improve its stability by encapsulating the perovskite inside the capillary. However, the high-quality perovskite film-coated hollow capillary fibers have yet to be realized. Here, we introduce a fast solvent exchange method which is used for the preparation of neat and smooth perovskite films deposited on the inner surface of capillary fibers. We demonstrate that this fast solvent exchange method is superior to the commonly used spontaneous diffusion-based precipitation method. The obtained hollow capillary fibers show a narrowed spectral width of 4.9 nm under pulse excitation due to the optical cavity effect. This new fabrication method can facilitate the development of perovskites in the fields of capillary lasing, microfluidic sensing, flexible LEDs and luminous fabrics.

## 1. Introduction

Metal halide perovskites have shown excellent optoelectronic properties, such as high absorption coefficient, controllable tunable spectrum, long-range carrier diffusion, high carrier mobility and low density of defect states [1,2,3]. Due to these merits, the external quantum efficiency of perovskite light-emitting devices (LEDs) has reached 23.4% [4], which is comparable with InP [5] and ZnTeSe [6] quantum dots-based LEDs. In addition, the small capture cross-section of defects and slow Auger recombination rates enable perovskites to realize amplified spontaneous emission [7] and lasing (pulsed [8,9] and continuous-wave [10,11,12,13]) with low thresholds at room temperature.

The stability of perovskites is, however, a long-standing challenging issue, especially under the conditions of high humidity, oxygen exposure and/or ultraviolet irradiation. [14,15,16] Capillary fibers, which offer a closed space, provide a feasible solution to this problem as sensitive perovskites can be sealed inside the capillary without being exposed to the environment. The annular structure of a capillary cross-section makes itself an optical microcavity which can support whispering-gallery mode (WGM) lasing when filled or coated with gain media and is compatible with microfluidic systems for biochemical sensing and detection, etc. [17,18,19].

In prior work, capillaries have often been used as ring- or disk-type cavities for light-emitting polymers. Yoshida et al. coated a layer of poly (3-alkylthiophene) in a microcapillary and demonstrated WGM lasing. They found the microcapillary structure was superior to a conventional micro-ring [20]. In addition, other well-defined WGM lasers [21] with ring-shape polymer films and Raman lasers based on dye-doped polymer films [22] have also been realized afterwards using capillaries. In contrast, there are few reports on the preparation of perovskites inside a capillary. Kurahashi et al. demonstrated that the capillary method can effectively protect air-sensitive perovskites by making the solution crystallize directly inside the microcavity and prepared WGM lasers with low thresholds [23]. However, using this method, instead of forming a thin layer on the inner surface of a capillary as often happened for polymers, the crystallized perovskites filled up the entire space inside the capillary, which not only consumed a large amount of perovskites, but also blocked the fluidic channel for microfluidic devices. More importantly, this method, which was based on spontaneous diffusion, has very limited control over the dynamic process of perovskite dispersion and crystallization. Therefore, it is important to find a more reliable method for repetitive preparations of perovskite film-coated hollow capillary fibers with simple processes and low costs, so that they can be applied in microfluidic sensing, lasers, flexible electronic fabrics, etc.

Here, in order to coat a high-quality thin layer of air-sensitive perovskites on the inner surface of a capillary fiber, we introduce a fast solvent exchange method. We compare this method with a traditional spontaneous diffusion-driven precipitation process and discuss the dynamics of perovskite precipitation. We find the fast solvent exchange method largely improves the film uniformity and smoothness and the prepared hollow capillary fibers can effectively narrow the spectral width of perovskites.

## 2. Materials and Methods

### 2.1. Micro-Fluid Assembly

Capillary fibers with diameters of 40 μm and 70 μm were obtained from Polymicro Technologies (Tempe, AZ, USA). Their polymer capping layers were removed by mild flaming to expose the transparent quartz micro-fluid. Unlike the previously reported densely packed WGM perovskite lasers that filled up the capillary [23], which could be obtained by making use of its spontaneous crystal growth and aggregation nature, critical issues arose as we intended to assemble the perovskite layer only on the inner surface due to the same crystal formation processes.

**Figure 1:**
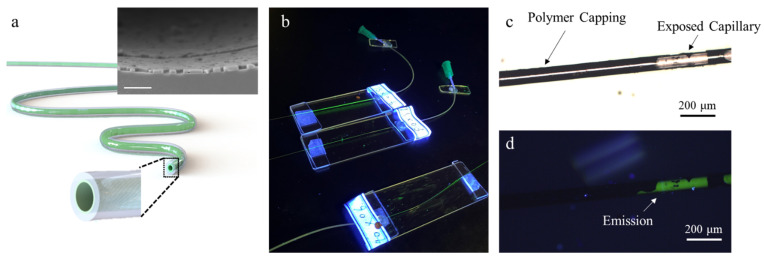
Luminescent perovskite micro-fluid. (**a**) A schematic diagram of the presented luminescent flexible capillary with inner surface coating. The inset shows compact and uniform perovskite assembly on the inner surface. Scale bar: 2 µm. (**b**) The experiment setup. Microscope image of the luminescent capillary under (**c**) white light and (**d**) UV lamp illumination, respectively.

In this regard, we propose a fast solvent exchange method that leads to instant crystallization in the capillary. A schematic diagram of the presented device is depicted in Figure 1a. The luminescent perovskite crystals (MAPbBr_3_) are deposited uniformly on the inner surface of the capillary, which is confirmed by the inset cross-section SEM image. As shown in Figure 1b, the capillary fiber is sealed with a flexible hose that is connected to a syringe at one end by NOA68 (Norland Products, Cranbury, NJ, USA) and has an open terminal at the other end. With such a setup, we are able to fill the solutions in by injection, drawing, or simple capillary force on demand. In principle, the solvent exchange deposition mainly includes three steps: (i) filling the capillary fiber by the perovskite precursor solution (described in the next section); (ii) drawing out the DMSO solvent (Shanghai Aladdin Biochemical Technology Co., Ltd., Shanghai, China) by its mutual solvent (solvents that have mutual solubility like water and alcohol) to start crystal precipitation; (iii) hot baking the capillary fiber to evaporate the remaining mutual solvents. To confirm that perovskite is successfully deposited on the inner surface, microscope images taken at the same region are shown in Figure 1c,d, under white light and ultra-violet lamp illumination. The opaque area in Figure 1c represents the polymer capping, while the exposed quartz capillary is transparent. Under UV lamp excitation, the green perovskite emission can be blocked by the passive polymer capping and is only observed where the quartz capillary is exposed, as shown in Figure 1d. This comparison clearly indicates that the perovskite crystals reside on the inner surface.

### 2.2. Neat Perovskite Film Assembly with NdBr_3_ Additives

**Figure 2:**
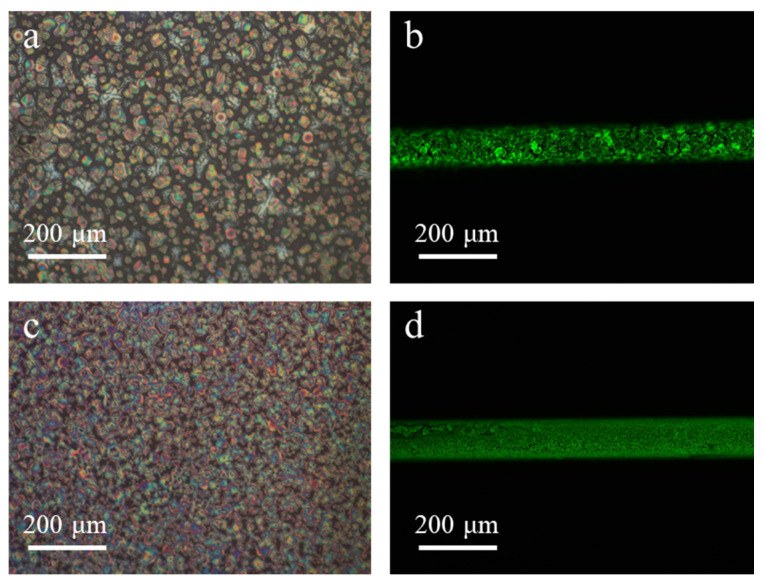
Neat film assembly with NdBr_3_ additives. (**a**) Spontaneous crystallization assembly on a planar substrate. (**b**) Film assembly in a capillary fiber without tactical control. (**c**) NdBr_3_ additives modified crystallization on a planar substrate. (**d**) Additive controlled film assembly in a capillary fiber.

The MAPbBr_3_ precursor solution is obtained by dissolving MABr (Xi’an Polymer Light Technology, Xi’an, China) and PbBr_2_ (Xi’an Polymer Light Technology, Xi’an, China) in DMSO with a molar ratio of 1:1.06, according to the desired concentrations. The solution is stirred for 4 h at 60 °C and then filtered by 0.22 µm PTFE (Polytetrafluoroethylene, REBIO, Shanghai, China) to remove the remaining sediments. As described earlier, the ring-shaped active structure is preferred to be smooth and well defined in order to make full use of the capillary structure. However, the spontaneous crystallization during film formation differentiates perovskite from organic light-emitting materials. Figure 2a shows a typical MAPbBr_3_ film without tactical control on its spontaneous crystallization process. The precipitated tiny crystals tend to aggregate to form separately dispersed large crystallites, with sizes up to tens of micrometers that are comparable to the capillary radius, as shown in Figure 2b. Such behavior will be detrimental in optical microstructure applications. For this reason, Kurahashi et al. filled the whole capillary by controlled single-crystal growth under small diameter conditions [22].

On the other hand, we previously reported a trivalent-neodymium (NdBr_3_) additive modulated MAPbBr_3_ perovskite nucleation and growth method for the formation of smooth perovskite films [24]. The presence of NdBr_3_ nuclei significantly suppressed the size of MAPbBr_3_ crystallites, which is beneficial for the formation of smooth perovskite films in capillary fibers. With 5% NdBr_3_ additives, Figure 2c,d present the spontaneously formed crystal films on a planar substrate and in a capillary fiber, respectively. It can be seen that not only the size of crystallites is suppressed to a large extent, but they also bridge together toward a neat film. These results pave the way for the formation of neat films on the inner surface of a capillary fiber.

### 2.3. Solvent Exchange Perovskite Film Deposition Methods

**Figure 3:**
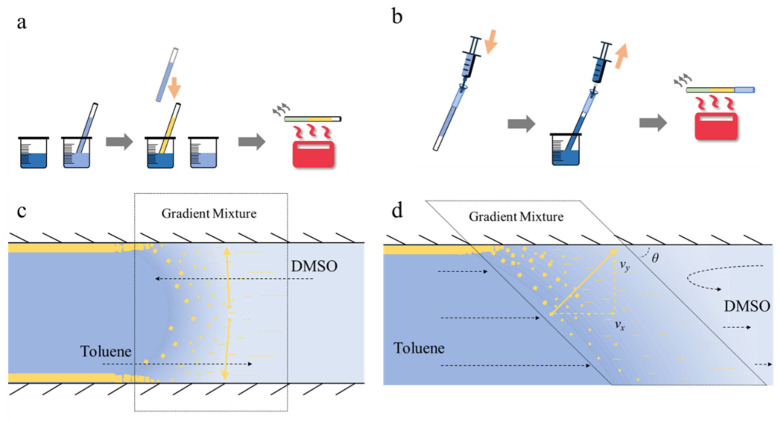
Solvent exchange film assembly methods. (**a**) A dipping method incorporating the spontaneous solvent exchange crystal forming and deposition. (**b**) The fast solvent exchange film assembly incorporating external force. (**c**,**d**) The proposed physical pictures for the respective methods. With a fluid velocity in the *x*-direction, the crystal deposition direction is rotated by *θ*.

In order to obtain a uniform perovskite inner coating, a dipping method and a syringe assisted force driving method are explored. They are depicted in Figure 3a,b, respectively. 

In a diffusion modulated dipping solvent exchange process, no external force is applied to accelerate the solvent flow in the capillary. The suspended capillary terminal is first dipped in the perovskite precursor solution for precisely 15 s, enabling the filling of precursor solution by capillary force. The same faucet is then swiftly immersed in toluene solution and the solvent exchange process begins spontaneously. The transparent quartz capillary turns brown quickly and becomes luminescent under UV excitation. After 10 s, the capillary is transferred to a 90 °C hotplate and dried for 4 h to remove the remaining toluene. This automatic process is indicated in Figure 3c. Since toluene and dimethyl sulfoxide (DMSO) are mutual solvents, they diffuse into each other naturally upon direct contact, forming a gradient mixture that propagates along the capillary as diffusion develops. Meanwhile, MAPbBr_3_ is not soluble in toluene; thus, a precipitation region exists in this gradient mixture where perovskite crystals form and grow, as shown in Figure 3c. 

In an external force driving method, as shown in Figure 3b, the perovskite precursor solution is injected to fill up the capillary. Then, the other terminate of the capillary is immersed in the toluene solution and a new syringe quickly draws the toluene in until a considerable amount enters the syringe to remove all remaining precursor solution. This solvent exchange initiates fast perovskite crystal precipitation instantly and the transparent quartz capillary turns translucent upon toluene inflow. After this, the capillary is heated on a hotplate likewise and a uniform micro-fluid with perovskite inner coating can be obtained.

Since the toluene has been drawn into the capillary, the film formation process should be different. In this case, obviously, not all MAPbBr_3_ will be deposited on the inner surface, as a considerable amount of the precursor solution will be drawn out before solvent exchange in the capillary. A simplified demonstration is shown in Figure 3d. While the diffusion modulated solvent exchange forms the critical gradient mixture as in Figure 3c, the externally driven flow applies an additional *v*_x_ on the crystal deposition direction, forming an angle *θ* between the transition region and the inner surface, as indicated by the yellow dashed line. As the transition region develops along the capillary fiber driven by the external force, perovskite crystallites are deposited on the inner surface.

## 3. Results and Discussion

### 3.1. Diffusion Modulated Solvent Exchange

**Figure 4:**
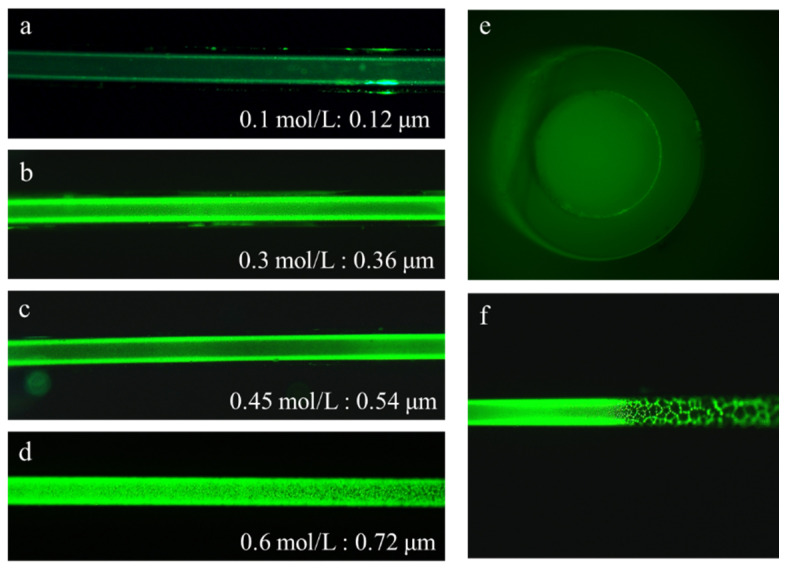
Diffusion modulated solvent exchange film assembly results. (**a**–**d**) Film assembly results incorporating different precursor concentrations. (**e**) Microscope cross-section image. (**f**) Sparsely dispersed large crystallites due to the slowed solvent exchange rate as the distance from the faucet increased to a large extent.

The results obtained from the dipping method are shown in Figure 4a–d, with varied concentrations of precursor solutions. In a spontaneous diffusion modulated solvent exchange precipitation process, by assuming all MAPbBr_3_ components along ∆*x* in the capillary contribute to the assembled film on the inner surface, a rough estimation of film thickness *d* can be made by:

*d* = (*r* × *mM*)/(2*ρ*) (1)

where *r* is the capillary radius, *m* is the molar concentration, *M* is the molar mass and *ρ* is the mass density approximated using the parameters obtained from perovskite crystal structure as 3.93 g/cm^3^. These results are given in the respective Figures. Considering that the actual crystal size should be a synergistic effect of precursor concentration and growth time, the dense-pack condition can be found when the actual crystal size matches the estimated film thickness. The crystal size is measured to be around 1 μm at 0.6 mol/L by magnifying the cross-section 1000×. This explains the sparse distribution at 0.1 mol/L and the nearly compact film at 0.6 mol/L. Relatively uniform results are obtained from 0.3 mol/L to 0.45 mol/L, possibly due to a smaller crystal size compared with the 0.6 mol/L cross-section sample.

However, the utterly spontaneous diffusion-driven precipitation process suffers from the fluctuations in actual microscopic conditions, as shown typically in Figure 4f, especially at high concentrations. For instance, as the distance from the dipping faucet increases, the solvent exchange rate should slow down. Thus, a tiny crystal undergoes more Brownian motions to merge with other crystals and form a large crystallite, leading to the sparsely dispersed large crystallites in Figure 4f. In addition, the capillary force driven solvent filling also limits the length of obtained luminescent micro-capillaries. Hence, a more controllable method should be sought. 

### 3.2. Flow Velocity Controlled Solvent Exchange

**Figure 5:**
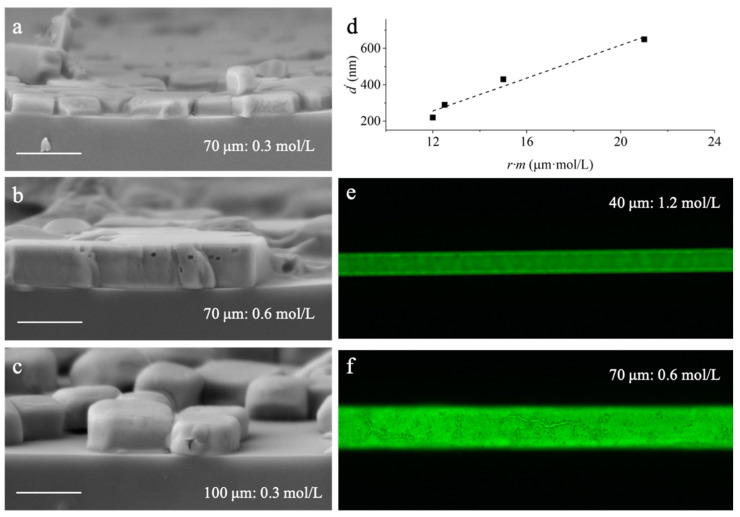
Flow velocity controlled uniform solvente. (**a**–**c**) Cross-section SEM images of inner-surfaces of perovskite-coated capillaries with varied capillary diameters and concentrations of precursor solutions. Scale bar: 1 µm. (**d**) Linear thickness dependence described by Equation (2). (**e**,**f**) The film assembly results applying diameter-optimized precursor concentrations.

To overcome the non-uniformity and variations in the spontaneous diffusion modulated solvent exchange method, a reliable syringe assisted external force driving fast solvent exchange method is developed, as described in Figure 3b and Section 2.3. The film assembly results are presented in Figure 5a–c. An optical image showing the uniform film assembly results along the whole capillary can be found in Figure 1b. Film thicknesses obtained by the cross-section SEM images show a similar linear dependence on *r × m* described by Equation (1), but the slope *k* = 45 ± 5 becomes considerably smaller than *M*/2*ρ* = 61 due to the difference in crystal deposition depicted in Figure 3d.

Since a fluid velocity along *x*-direction is imposed on the growing crystal, the observed smaller *k* constant can be ascribed to the rotated deposition direction *θ.* Previously, the perovskite components in volume $\pi r^{2}\times \Delta x$ should spontaneously move to the lateral area 2πr×Δx leading to Equation (1). However, with the introduction of *v*_x_, the considered cross-section $\pi r^{2}$ is distorted to be $\pi r^{2}/\sin\theta .\;$ In this regard, if *N* crystals are formed at the transition region cross-section by a spontaneous process, they are now dispersed in a larger area $\pi r^{2}/\sin\theta$. Thus, the thickness *d′* should be approximated including the effect of *θ*:

*d′* = *d* × sin*θ* = *r* × *m* sin*θ M*/2*ρ* (2)

where *d* is the estimation of film thickness from Equation (1), *d′* is the thickness modulated by *θ*. Incorporating the slope of Figure 5d, the value of *θ* is determined as 48°. This result will serve as guidance for future efforts in improving the film morphology and exploring its wide luminescent capillary applications. Figure 5e,f show the optical images of the fabricated capillaries with similar *r × m*, which are much more compact and uniform compared with the results presented in Figure 4.

### 3.3. Structure Dependent Emission

**Figure 6:**
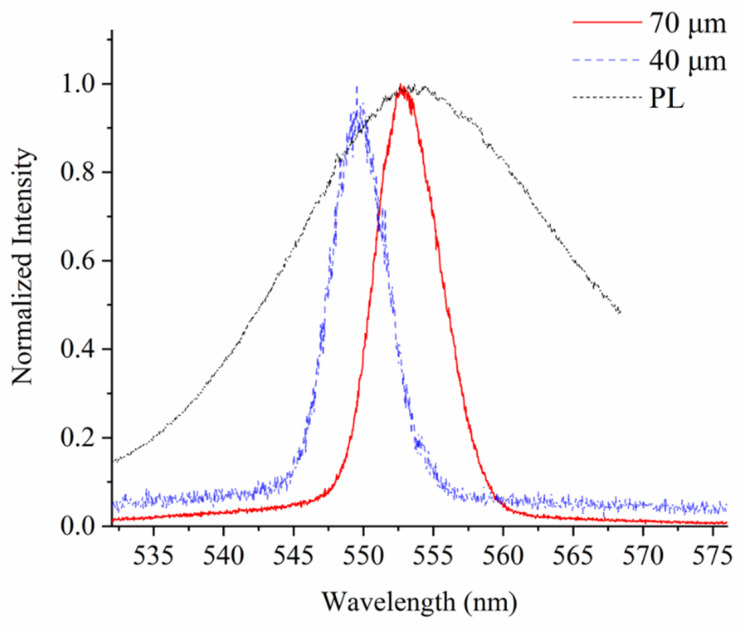
Structure dependent narrow emission. The FWHMs reduce to 5.5 nm (in red) and 4.9 nm (in blue) for the 70 µm and 40 µm sample, respectively, at high pump power exceeding 7.2 μW, indicating good film morphology for the ring optical structure emission enhancements. PL spectrum (in black) was recorded by pumping a sample below threshold without Nd additives.

To further evaluate its potential in optical applications, the optimized samples are excited by a nanosecond pulsed laser (Opotek, Vibrant 355 II, Carlsbad, CA, USA) delivering 410 nm pump pulse with a repetition rate of 10 Hz. At high pump rates, the emission spectrums in Figure 6 show significant full-width half-maximum (FWHM) reductions from 25 nm to 5.5 nm and 4.9 nm for the 70 µm and 40 µm samples, respectively. Such narrow emission resembles the amplified spontaneous emission behavior of planar perovskite films, which indicates apparent optical cavity effects and small round-trip optical loss in the capillary. The emission from the 40 µm sample shows a blue-shift of 3.3 nm, as the long-wavelength modes are more prone to curvature due to the larger incident angles at the boundaries. These observations not only indicate its potential application in capillary lasing and flexible light-emitting fibers but also provide additional support for the claim of neat, smooth inner surface luminescent films.

## 4. Conclusions

In conclusion, we used the MAPbBr_3_ solution with NdBr_3_ to realize the nucleation and growth of perovskite crystals and assembled the perovskite layer only on the inner surface of a capillary fiber, leaving enough space for microfluidic applications. A fast solvent exchange method was proposed to replace the spontaneous diffusion modulated solvent exchange method (a dipping method) and we have effectively prepared neat, smooth luminescent perovskite films on the inner surface of capillary fibers by solving the problems of non-uniformity and variations. Prepared with this method, the thin perovskite films with a narrowed linewidth (from 25 nm to 4.9 nm) show apparent optical cavity effect, which indicates its potential for applications in capillary lasing, microfluidic sensing and flexible electronic fabrics.

## Data Availability

The data presented in this study are available on request from the corresponding author.
